# Organizational access points and substance use disorder treatment utilization among Black women: a longitudinal cohort study

**DOI:** 10.1186/s40352-023-00236-7

**Published:** 2023-08-21

**Authors:** Sugy Choi, Amanda Bunting, Talia Nadel, Charles J. Neighbors, Carrie B. Oser

**Affiliations:** 1 Department of Population Health, New York University Grossman School of Medicine, New York, NY USA; 2 Department of Sociology, Center on Drug & Alcohol Research, Center for Health Equity Transformation, University of Kentucky, Lexington, KY USA

**Keywords:** Criminal justice system, Touchpoints, Substance use disorder, Health disparities, Longitudinal analysis

## Abstract

**Introduction:**

Health and social service organizations, including the emergency department (ED) and public assistance programs, constitute a social safety net that may serve as an “access point” for substance use treatment utilization. Racialization of substance use disorder (SUD) and gender disparities in access to treatment contribute to differences in health and social service utilization, including substance use treatment for Black women. We therefore explored the role of various access points in facilitating the use of substance use treatment among Black women with substance use and involvement in the criminal justice system.

**Methods:**

We used data from the Black Women in the Study of Epidemics (B-WISE) project (2008–2011), which recruited Black women who use drugs from community, probation, and prison recruitment settings in Kentucky. B-WISE is a three-wave panel survey collected on a six-month interval. We estimated dynamic panel models to understand whether time-varying use of services influenced women’s substance use treatment utilization over 18-months, adjusting for time-invariant characteristics. We stratified the analysis based on where women were recruited (i.e., community, prison, and probation).

**Results:**

The sample included 310 persons and 930 person-waves. For the community and prison samples, the use of an ED in the 6 months prior decreased women’s likelihood of subsequent substance use treatment use (Coef: -0.21 (95% CI: -0.40, -0.01); -0.33 (95% CI: -0.60, -0.06), respectively). For the probation sample, receiving support from public assistance (i.e., food stamps, housing, cash assistance) increased the likelihood of subsequent substance use treatment use (0.27 (95% CI: 0.08, 0.46)).

**Conclusion:**

Interactions with health and social service organizations predicted Black women’s use of substance use treatment services and varied based on their involvement in the criminal justice system. Public assistance venues for Black women on probation may be a point of intervention to increase their access to and use of substance use treatment.

## Background

Gender differences in substance use, substance use disorder (SUD), and utilization of health services, including SUD treatment, have become widely recognized (McHugh et al., 2018). Engaging women who have SUD in substance use treatment can be particularly challenging (Amaducci et al., 2020; Greenfield et al., 2007; Pinedo et al., 2020). Women often face barriers to treatment, like childcare responsibilities, that extend beyond what is traditionally considered to be the responsibility of the health sector and also must contend with gender-specific social stigma relating to drug use including the criminalization of substance use during pregnancy and assumptions of child maltreatment (Greenfield et al., 2010). While not all substance use requires treatment, individuals with the clinical diagnoses of SUDs, would benefit treatment. Although only a small fraction of people of all genders with SUD utilize SUD treatment services, this discrepancy is even more stark when looking at women in particular (Greenfield et al., 2010). In the face of these disparities, gender-specific SUD treatment strategies have evolved, but access to tailored care remains limited (Substance Abuse and Mental Health Services Administration, 2021).

It is crucial to adopt an intersectional lens that acknowledges the unique challenges faced by Black women when examining SUD treatment utilization among women. Structural racism and historical policies have contributed to the overrepresentation of Black women in the penal system (Bailey et al., 2017; Dettlaff & Boyd, 2020; Hill, 2004). Consequently, these women encounter additional hurdles due to their intersecting marginalized identities (Link & Oser, 2018; Perry et al., 2012, 2013, 2016; Redmond et al., 2020). Historically, the “War on Drugs” mentality and its’ both punitive and empirically ineffective enforcement practices have disproportionately negatively affected Black mothers in the United States (Chin, 2013; Cloud and Davis, 2015). Black women were at the focus of many drug control policies, but these oftentimes stigmatizing and punitive approaches have not translated into access to SUD treatment services for this population, particularly those who have a history of involvement with the criminal justice system (Thompson et al., 2016). Understanding the impact of criminal justice status on treatment utilization among this population is therefore pivotal.

Women who use substances navigate multiple siloed systems of care, such as SUD treatment, health care, mental health systems, social welfare systems, and the criminal justice system. Multiple health and social service organizations ─ including the emergency department (ED) and social services agencies that offer public assistance ─ that may act as referral settings were identified in a recent systematic scoping review (Choi, Rosenbloom et al., 2021). These settings constitute a social safety net and may serve as an “access point” for SUD treatment referrals.

To better understand the factors influencing SUD treatment utilization among Black women, Pescosolido’s Network Episode Model (Pescosolido, Gardner et al., 1998) offers insights into the complex social ties and the information, advice, and support shared in social networks that substantially influence how people make sense of their health concerns and the action they take. Those ties are dynamic and include an individual’s ties to public assistance or organizations in addition to social relationships that can lead them to health services (Pescosolido, Wright et al., 1998). Applying this model to understand the potential role of dynamic engagement with access points and their role in facilitating SUD treatment utilization among Black women can shed light on missed opportunities for culturally-tailored outreach and referral efforts.

The lack of progress in SUD treatment engagement can be tied to the longstanding underinvestment in health disparities and minority research (Blanco et al., 2022; Hall et al., 2022). Examining treatment use among Black women who use substances by women’s criminal justice backgrounds is a way to reevaluate disparities among this population and improve current strategies for prevention and treatment. In this paper, we sought to examine the role of prior service use (varying types of health, social, and criminal justice involvement) and use of SUD treatment services over time among Black women who use substances. We also stratified the analysis by women’s criminal justice backgrounds via their recruitment settings (i.e., community, prison, and probation). The Black Women in the Study of Epidemics (B-WISE) study dataset, described below, is particularly well-suited to exploring this topic due to its (1) longitudinal panel wave design; (2) comprehensive information on Black womens’ substance use history, treatment history, use of a wide array of health and social services, and history of involvement with the criminal justice system; and (3) incorporation of three different types of criminal justice backgrounds.

This study seeks to answer the following research questions: (1) What are the characteristics and differences in demographic, socioeconomic, and substance use-related variables among Black women in different criminal justice contexts (community, prison, and probation)? (2) What are the patterns and variations in the utilization of health and social services, as well as SUD treatment, among Black women in different criminal justice contexts over time? (3) How do various factors, including prior addiction treatment, health services utilization (such as doctor’s visits and emergency room visits), social services utilization (such as receipt of public assistance and disability benefits), and criminal justice system involvement (such as prior arrest), influence the utilization of SUD treatment among Black women in different criminal justice contexts over time?

## Methods

### Study population and data

We performed a secondary analysis on data from a study that collected longitudinal survey data in Kentucky between 2008 and 2011 using a wave system (three waves every 6 months over 18 months from time of recruitment per participant) to examine the relationship between Black women’s drug use, their involvement with the criminal justice system, and their health outcomes, including disparities (Pullen & Oser, 2017). To achieve this aim, the Black Women in the Study of Epidemics (B-WISE) study used a stratified sampling design with the goal of equally enrolling self-reported drug using and non-drug using populations, and recruiting proportionately from prison (35.6%), probation offices (31.5%), and community (32.9%) settings. However, given the high prevalence of drug use in the prison population as a whole, women who use drugs were over-represented (78%) in the B-WISE prison sample (Oser, 2015). While 643 total women were interviewed at baseline, due to the nature of our research question, our analysis was only performed on those women who both identified as persons who use drugs at baseline and participated in all waves of the 18-month follow-up data collection process (n = 310).

In addition to being willing to participate, all women were required to (1) self-identify as Black, (2) be at least 18 years of age, and (3) speak English in order to be eligible for the B-WISE study. In this longitudinal study, eligibility for participation was determined at baseline. Women who participated in the screening process were categorized into one of two groups based on their self-reported illicit drug use within the past year (or year prior to incarceration for women in the prison sample). There was an additional eligibility criteria for each sample. Specifically, women from the community sample were required to have no current or pending charges, upcoming cases, or court dates in order to be eligible for the study. However, it should be noted that over the course of the study, participants had the potential to become involved in the criminal justice system. Likewise, women in the prison sample were eligible for inclusion in the study if they were currently incarcerated and deemed eligible for release within 60 days of the baseline interview. (Pullen & Oser, 2017). Women in the probation sample had the additional eligibility criteria of currently being on probation.

Recruitment varied by the three groups. Women from the community sample were recruited via publicly posted (e.g. grocery stores, churches, bus stops, beauty salons) flyers and newspaper advertisements, which were targeted to areas with the highest proportions of Black residents based on Census data (Oser, 2015; Pullen & Oser, 2017). Women in the prison sample were recruited from three women’s prisons. All women in these prisons who were eligible for release within 60 days (based on lists provided by the Kentucky Department of Corrections) were mailed letters and invited to attend study informational sessions (Pullen & Oser, 2017). The probation sample was recruited from the waiting rooms of seven probation offices within the Kentucky Department of Corrections, with trained Black study interviewers approaching women during probation report days. Eligible participants were screened for drug use and willingness to participate, and those who qualified were scheduled for baseline interviews and testing procedures at a separate private location (Oser, 2015).

Women responded to surveys in 6-month increments (baseline, Wave 1 at 6 months, Wave 2 at 12 months, Wave 3 at 18 months). For women who were recruited from prisons, Wave 1 surveys were administered 6 months following their release from prison rather than 6 months from baseline (Pullen, 2014). The surveys were administered by trained Black women interviewers who recorded responses using computer-assisted personal interviewing software (CAPI) (Oser et al., 2016). In the total sample (both drug using and non-drug using populations), the response rates at each wave were 94%, 92%, and 90%, respectively (Pullen, 2014). Aside from baseline surveys for the prison population, which were conducted in prison visitation rooms, all baseline and follow-up surveys were conducted in private rooms at public locations such as libraries, university research offices, and community based organizations (Pullen & Oser, 2017). No prison or probation staff were present for any interviews. Additional methodological details are available elsewhere (Oser, 2015; Oser et al., 2011, 2016; Perry et al., 2012, 2013). The B-WISE study was approved by the University of Kentucky’s Institutional Review Board and a federal Certificate of Confidentiality was obtained from the National Institutes of Health.

### Measures

#### Dependent variables

We measured SUD treatment engagement across the three waves. Women were asked if they had participated in drug or alcohol treatment. Treatment services included: outpatient, residential, detox, or methadone treatment programs. Responses were coded such that 1 = use of treatment services in past 6-months and 0 = no use in the past 6-months.

#### Time-varying regressors

We used five binary variables for women’s use of doctor’s office or private health clinics, emergency room, receipt of public assistance (food stamps, housing assistance, AFDC, or TANF), disability benefits, and arrest in the past 6 months and created lag variables for each. Questions from the Miami Health Services Utilization (MHSU) measures were used (Chitwood et al., 1999; Oser, 2015). Since we were interested in exploring the effects of service use as possible referral sources for SUD treatment, using lag variables allowed us to account for the time it takes for referrals to happen.

#### Time-invariant socio-demographic and clinical regressors

Socio-demographic variables were drawn from the baseline survey data. We coded for age, educational attainment (0 = Less than High School;1 = High School [or GED equivalent]; and 2 = College or more), marital status (1 = Legally married; 2 = Single, 3 = never married; and 4 = Other [divorced, widowed, etc]), insurance type (1 = Medicaid; 2 = Other [employer provided health insurance, personal health insurance other than Medicaid, Medicare, VA/CHAWUS, or other]; and 3 = Not insured), frequency of any drug use (1 = less than weekly, 2 = more than weekly) and household income (1 = Less than $4,999; 2=$5000 to $9,999; 3=$10,000 to $19,999; 4=$20,000 to $29,999; 5=$30,000 to $39,999; 6 = More than $40,000; and 7 = Don’t know). The questions posed to assess drug-related problems were as follows: (baseline) “In the past year before incarceration, which drug caused the most serious problem?“ and (follow-up) “In the 6/12/18 months after your release, which drug caused the most serious problem?“ Primary substance that caused most serious problem was used to create dichotomous variables (Alcohol (0,1); Marijuana (0,1); Cocaine (0,1); and Opiates (0,1). Frequency of any drug use was coded as 1 = less than weekly and 2 = more than weekly.

### Statistical analysis

Descriptive statistics for baseline characteristics are presented by criminal justice status in Table 1. The chi-square test was used to compare the characteristics of the three groups: community, prison, parole. The percentage of women utilizing various services over time is presented by criminal justice involvement status at baseline in Tables 2 and 3. We assessed the significant differences across waves, by obtaining the coefficient on the variable “wave 3” and its associated robust standard error. This coefficient represents the estimated difference between the waves 1 and 3, while the robust standard error accounts for clustering effects. In Table 4, we employed a maximum likelihood estimator implemented with structural equation modeling to analyze the panel data spanning three waves. This method has been known to be less biased than generalized method of moments (Allison et al., 2017; Hamaker et al., 2015). This method allows for the adjustment of time variant and invariant characteristics, and error variances can also vary with time. In smaller samples, the potential for bias becomes more pronounced, making it crucial to choose an estimation method that offers better accuracy and reduced bias (Allison et al., 2017; Hamaker et al., 2015). In the first model, we included time-variant predictors and in the second model, we adjusted for time-invariant variables in addition to time-variant predictors from the previous model. All of the models clustered standard errors to minimize any potential problems of serial correlations and heteroscedasticity. We also estimated separate models for each recruitment population, since participant characteristics varied significantly.

**Table 1:** Participant characteristics by criminal justice status at baseline

Identified as person who uses drugs at baseline, no missing n	Community	Prison	Probation	Total	p-value
Age† (mean)	33.6	36.3	34.0	34.7	0.004
Household income (in year), %					0.465
Less than $4,999	31.7	31.4	23.1	29.0	
$5,000 to $9,999	17.8	16.1	22.0	18.4	
$10,000 to $19,999	31.7	21.2	27.5	26.5	
$20,000 to $29,999	5.9	9.3	9.9	8.4	
$30,000 to $39,999	5.0	10.2	6.6	7.4	
More than $40,000	6.9	8.5	5.5	7.1	
Don’t know	1.0	3.4	5.5	3.2	
Educational attainment, %					0.002
Less than High School	27.7	47.5	42.9	39.7	
High School (or GED equivalent)	24.8	30.0	27.5	27.4	
College or more	47.5	22.9	30.0	32.9	
Marital status, %					0.021
Legally married	7.9	17.0	8.8	11.6	
Single, never married	71.3	54.2	73.6	65.5	
Other (divorced, widowed, etc.)	20.8	28.8	17.6	22.9	
Primary substance that caused most serious problem, %					0.001
Alcohol	18.8	13.6	6.6	13.2	
Cannabis	22.8	20.3	22.0	21.6	
Cocaine	23.8	50.9	34.1	37.1	
Opiates	1.0	5.1	2.2	2.9	
Frequency of use, %					< 0.001
Less than weekly	22.8	4.2	14.3	13.2	
More than weekly	77.2	95.8	85.7	86.8	
Health insurance status, %					0.260
Medicaid	32.7	44.1	38.5	38.7	
Other	22.8	21.2	15.4	20.0	
Not insured	44.6	34.8	46.2	41.3	
N	101	118	91	310	

Note: †n = 306.

## Results

Table 1 displays significant differences on baseline characteristics across the three criminal justice status groups. Out of the women who completed the 18-month follow-up data collection process, 101 women from the community sample, 118 women from the prison sample, and 91 women from the probation sample identified as persons who used drugs at baseline. We observed statistically significant differences in age (p = 0.004), educational attainment (p = 0.002), and the primary substance that caused the most serious problem (p = 0.001) across the community, prison, and probation samples. The mean age of participants in was 34.7 years. The mean age for the community sample was 33.6 years, for the prison sample it was 36.3 years, and for the probation sample it was 34.0 years. The majority of participants in the community sample had a college degree or higher (47.5%), while a higher proportion of participants in the prison sample had less than a high school education (47.5%). The most common primary substance that caused the most serious problem in all three groups was cocaine, but the percentage of women reporting this in the prison sample (50.9%) was twice as high as the percentage of women reporting this in the community sample (23.8%). Women recruited from probation offices were least likely (6.6%) to report alcohol as the substance that caused the most serious problem for them compared to those recruited from the community (18.8%) and from prisons (13.6%). Women recruited from the prison setting were more likely to be married, have a household income above $20,000 per year, and have less than a high school education. The majority of women were either on Medicaid (38.7%) or uninsured (41.3%).

Women in the prison and probation samples were more likely than women in the community sample to have had a doctor’s visit in the past 6 months at each of the three waves. Across all samples and waves, women from the prison sample at Wave 1 had the highest percentage of ED visits in the past 6 months (11.9%). As noted above, Wave 1 survey data for this sample corresponds to the six-month period following their release from prison. Across all three waves, women from the community sample were least likely to have received disability benefits in the past 6 months. In order to be eligible for the study, women from the community sample could not have any current or pending criminal justice involvement at the time of recruitment. However, at Waves 1, 2, and 3 respectively, 10.9%, 8.9%, and 11.9% of them had been arrested within the past 6 months. Across all samples and waves, the majority of women had received some form of public assistance within the past 6 months. The results indicate that there were no significant differences in the percentage of participants who had a doctor’s visit or emergency department (ED) visit in the past 6 months across the waves. However, a significant difference was found in the utilization of social services, in the receipt of public assistance (p = 0.006), specifically among the prison and probation sample (p = 0.025; 0.007) (Table 2).

**Table 2:** Organizational ties across time by criminal justice status

	Wave 1 %	Wave 2 %	Wave 3 %	p-value
Health services				
Doctor’s visit in the past 6 months	36.0	37.1	38.1	0.478
Community	29.7	30.7	31.7	0.696
Prison	36.4	39.8	42.4	0.275
Probation	40.7	41.8	38.5	0.716
ED visit in the past 6 months	9.9	5.7	6.7	0.097
Community	9.9	4.0	5.0	0.138
Prison	11.9	7.6	9.3	0.469
Probation	7.7	3.3	5.5	0.483
Social services				
Received public assistance (food stamps, housing assistance, TANF, etc.) in the past 6 months	58.8	57.1	67.3	0.006
Community	62.4	53.5	61.4	0.862
Prison	57.6	55.9	70.3	0.025
Probation	55.0	61.5	70.3	0.007
Received disability benefits in the past 6 months	12.6	12.0	15.4	0.071
Community	5.9	5.9	5.0	0.566
Prison	18.6	17.8	24.6	0.162
Probation	9.9	8.8	15.4	0.096
Criminal justice system				
Arrested in the past 6 months	15.4	13.5	13.1	0.293
Community	10.9	8.9	11.9	0.809
Prison	18.6	16.1	13.6	0.291
Probation	18.7	14.3	14.3	0.417
N	310	310	310	

Note: Community (n = 101); Prison (n = 118); Probation (n = 91)

Despite experiencing an overall decrease in SUD treatment utilization over time (p = 0.004), the prison sample had higher treatment utilization than either the community or probation samples at each of the three waves. This seems to be driven by a significant reduction (16.1%) in SUD treatment utilization for the prison group across waves (p = 0.002) (Table 3).

Table 4 presents the cross-lagged panel model results. Prior ED visit (i.e., lagged variable) significantly negatively predicted SUD treatment for Black women, especially for community and prison samples (Coef: -0.21 (95% CI: -0.40, -0.01); -0.33 (95% CI: -0.60, -0.06), respectively). For the probation sample, receiving support from public assistance (i.e., food stamps, housing, cash assistance) and SUD treatment use in the prior 6 months increased the likelihood of subsequent SUD treatment use (0.27 (95% CI: 0.08, 0.46); 0.36 (95% CI: 0.01, 0.70), respectively).

**Table 3:** Substance use disorder treatment utilization by criminal justice status across 18 months

	Wave 1 %	Wave 2 %	Wave 3 %	p-value
Total	28.9	23.9	20.8	0.004
Community	10.9	8.9	7.9	0.409
Prison	47.5	39.0	31.4	0.002
Probation	25.3	20.9	22.0	0.534
N	310	310	310	

Note: Community (n = 101); Prison (n = 118); Probation (n = 91)

Women who were divorced (or widowed) were more likely to engage in SUD treatment compared to married women (0.15 (95% CI: 0.04, 0.27)). Women using opioids or cocaine were more likely to engage in SUD treatment (0.19 (95% CI: 0.08, 0.29); 0.44 (95% CI: 0.22, 0.66), respectively) compared to those who listed alcohol or cannabis as the primary substance that caused the most serious problem for them. Overall, prison samples were more likely to engage in SUD treatment compared with community samples (0.15 (95% CI: 0.05, 0.24)).

**Table 4:** Dynamic panel models predicting SUD treatment utilization

	Total		Community		Prison		Probation	
Model	**(1)** **Partially adjusted**	**(2)** **Fully adjusted**	**(1)** **Partially adjusted**	**(2)** **Fully adjusted**	**(1)** **Partially adjusted**	**(2)** **Fully adjusted**	**(1)** **Partially adjusted**	**(2)** **Fully adjusted**
Time variant variables								
Health Services								
Prior addiction treatment	0.26* (0.03, 0.48)	0.23* (0.03, 0.43)	0.21 (-0.10, 0.52)	0.01 (-0.20, 0.22)	0.05 (-0.23, 0.33)	0.01 (-0.24, 0.26)	0.36* (0.01, 0.70)	0.22 (-0.06, 0.51)
Prior doctor’s visit	-0.04 (-0.14, 0.07)	-0.04 (-0.14, 0.07)	-0.04 (-0.19, 0.10)	-0.07 (-0.18, 0.04)	-0.03 (-0.20, 0.13)	-0.03 (-0.20, 0.13)	-0.07 (-0.26, 0.12)	-0.05 (-0.23, 0.13)
Prior ED visit	-0.24* (-0.41, -0.06)	-0.24* (-0.41, -0.07)	-0.24* (-0.49, -0.00)	-0.21* (-0.40, -0.01)	-0.33* (-0.60, -0.05)	-0.33* (-0.60, -0.06)	-0.09* (-0.43, 0.25)	-0.11 (-0.43, 0.21)
Social Services								
Prior receipt of welfare or public assistance^a^	0.06 (-0.04, 0.15)	0.05 (-0.04, 0.15)	-0.03 (-0.17, 0.11)	-0.02 (-0.13, 0.09)	-0.01 (-0.17, 0.15)	-0.01 (-0.17, 0.14)	0.30* (0.10, 0.50)	0.27* (0.08, 0.46)
Prior receipt of disability benefits	-0.13 (-0.32, 0.06)	-0.12 (-0.31, 0.06)	0.09 (-0.21, 0.40)	0.09 (-0.15, 0.32)	-0.21 (-0.46, 0.04)	-0.20 (-0.45, 0.05)	0.03 (-0.41, 0.47)	0.07 (-0.35, 0.49)
Criminal Justice System								
Prior arrest	0.02 (-0.10, 0.14)	0.02 (-0.10, 0.14)	-0.06 (-0.26, 0.14)	-0.05 (-0.21, 0.11)	0.03 (-0.16, 0.23)	0.04 (-0.16, 0.23)	0.13 (-0.06, 0.33)	0.12 (-0.06, 0.31)
Time invariant variables (baseline characteristics)								
Age		0.00 (-0.00, 0.00)		-0.00 (-0.01, 0.00)		0.01 (-0.00, 0.02)		-0.00* (-0.01, 0.00)
Educational attainment								
Less than High School		Ref		Ref		Ref		Ref
High School (or GED equivalent)		-0.04 (-0.12, 0.04)		0.03 (-0.07, 0.13)		-0.14 (-0.30, 0.03)		-0.00 (-0.16, 0.16)
College or more		-0.03 (-0.12, 0.04)		0.05 (-0.05, 0.15)		-0.19 (-0.04, 0.01)		0.02 (-0.15, 0.87)
Marital status								
Legally married		Ref		Ref		Ref		Ref
Single, never married		0.08 (-0.02, 0.18)		0.06 (-0.08, 0.20)		0.04 (-0.16, 0.24)		0.11 (-0.10, 0.32)
Other (divorced, widowed, etc.)		0.15* (0.04, 0.27)		0.12 (-0.03, 0.26)		0.12 (-0.10, 0.33)		0.28* (0.02, 0.54)
Primary substance that caused most serious problem								
Alcohol		Ref		Ref		Ref		Ref
Cannabis		0.02 (-0.09, 0.13)		-0.01 (-0.12, 0.10)		-0.06 (-0.32, 0.20)		0.27* (0.01, 0.54)
Cocaine		0.19* (0.08, 0.29)		0.22* (0.11, 0.34)		0.17 (-0.06, 0.38)		0.29* (0.02, 0.55)
Opiates		0.44* (0.22, 0.66)		0.89* (0.48, 1.29)		0.33 (-0.04, 0.71)		0.92* (0.40, 1.43)
Health insurance status								
Not insured		Ref		Ref		Ref		Ref
Medicaid		0.02 (-0.06, 0.10)		0.31 (-0.07, 0.13)		0.01 (-16.9, 19.2)		-0.08 (-0.26, 0.0)
Other		-0.05 (-0.15, 0.05)		-0.03 (-0.14, 0.08)		-0.11 (-0.17, 0.19)		-0.14 (-0.35, 0.06)
Recruitment sites								
Community		Ref						
Prison		0.15* (0.05, 0.24)						
Probation		0.07 (-0.01, 0.15)						
Fit indices								
RMSEA	0.042	0.000	0.026	0.057	0.000	0.026	0.079	0.046
AIC	3375.60	8844.44	735.80	2269.34	1677.40	3638.31	964.97	2408.32
BIC	3741.78	10182.13	992.09	3072.18	1948.93	4488.91	1211.03	3179.15

**Notes**: ^a^(food stamps, housing assistance, TANF, etc.); RMSEA = Root mean squared error of approximation; AIC = Akaike’s information criterion; BIC = Bayesian information criterion

* significant at p < 0.05

## Discussion

Overall, SUD treatment utilization decreased over time, in line with prior studies that demonstrate low treatment retention rates, particularly among Black women (Redmond et al., 2020). Not surprisingly, the prison sample had higher treatment utilization than either the community or probation samples. According to the literature, large proportions of treatment referrals come from the criminal justice system and treatment is often a condition of parole (Weisner & Schmidt, 1995). Additionally, women who use substances are nearly three times as likely to have an incarceration history, compared with women who do not use substances (Choi et al., 2021a, b). The highest risk for overdose occurs within the first two weeks after a person’s release from prison highlighting the importance of linkage to treatment in the community (Binswanger et al., 2007, 2013).

We found that for Black women in the community and prison samples, prior ED visits decreased women’s likelihood of subsequent SUD treatment utilization. This negative association between ED use and SUD treatment utilization among Black women is not surprising. If women utilize EDs for non-emergency medical care, these same individuals may be less likely to have usual places of care, including SUD treatment. However, if EDs have been used for substance-related problems, including overdose, they can be an important source of referral for SUD treatment. There is a growth in the literature about the importance of linking ED patients to SUD treatment, especially for patients with opioid use disorder who may benefit from medications (Choi et al., 2019; Duber et al., 2018; Rockett et al., 2005; Sullivan et al., 2021). In addition, it is worth considering that the use of EDs among these women may serve as a “signal” indicating a higher severity of addiction or greater levels of disadvantage and poor health compared to those who have not recently sought ED care (Bogenschutz et al., 2014; D’Onofrio & Bernstein, 2015; Hawk & D’Onofrio, 2018; Moe et al., 2017; Sandoval et al., 2010; Sun et al., 2003). This suggests that ED use may reflect underlying factors that contribute to substance use.

For women in the probation sample, receiving support from public assistance (i.e., food stamps, housing assistance, cash assistance, etc.) increased the likelihood of subsequent SUD treatment. Social services may be acting as an access point for treatment for this population; it is also possible that the community supervision requirements and conditions such as close surveillance, frequent urine toxicology, or mandated treatment may explain this association. Community supervision programs often collaborate with social service agencies to provide comprehensive support to individuals, including SUD treatment. We did not find this association among the prison sample, which could point to the role of varying degrees of justice involvement and/or severity in affecting SUD treatment utilization. Several potential factors could contribute to the lack of a similar finding in the prison sample after their release. These factors may include differences in the characteristics and experiences of individuals in the post-prison phase compared to those on probation (Phelps et al., 2022). For example, the prison sample may have undergone different reentry processes, experienced varying levels of social support, or faced unique challenges related to employment, housing, or family reunification after release (Kulkarni et al., 2010; Oser et al., 2016; Van Olphen et al., 2009). Additionally, the availability and accessibility of SUD treatment services and support systems may vary between the probation and post-prison contexts.

Yet, similar to prior studies that reported higher use of public assistance among women who use substances compared to women who do not use substances (Choi et al., 2021a, b; Nelson-Zlupko et al., 1995), the majority of women in this sample also had received some form of public assistance within the past 6 months. Although the majority used social services in the community and prison samples, these were not effective access points for women to utilize SUD treatment services. This may be because women convicted of certain crimes may not be eligible for some of these social services (Allard, 2002; Sohoni & Piatkowska, 2022; Van Olphen et al., 2009). Discriminatory policies related to some drug-related offenses can be barriers to accessing social and health services. Additionally, this finding may reflect how women fear having their benefits taken away since drug toxicology and/or asking for linkage to SUD treatment can disqualify people with a criminal justice history from an array of social service benefits (McCarty et al., 2012). The lack of engagement suggests that there is a need for broadening outreach efforts beyond the health care system (Pollack & Reuter, 2006; Schmidt & McCarty, 2000). Innovative approaches are needed to engage women in SUD treatment in agencies they are most likely to frequent. There is a need to understand how to integrate services to meet the social and treatment needs of women without stigma.

Additionally, prior SUD treatment increased women’s likelihood of future treatment utilization for women in the probation sample, which is encouraging considering SUD is a chronic health condition (McLellan, 2002; McLellan et al., 2000) often requiring multiple treatment episodes (Oser et al., 2011). Prior health service utilization did not increase the likelihood of referrals to SUD treatment. Efforts are also needed in health care settings to find ways to decrease stigma and increase screening and referral efforts to route women who need treatment services to the appropriate level of subsequent care. Barriers like stigma and racism at each of the respective access points may exacerbate issues navigating to SUD treatment services for this historically underserved group.

Women’s engagement in treatment also varied by the type of substances used. Women who cited that opioids or cocaine caused the most problems for them were more likely to engage in treatment, while women with problems with alcohol and cannabis were not likely to engage in treatment. These findings are interesting, as we have evidence-based medications for alcohol and opioid use but limited evidence-based options available for cannabis and cocaine. Further, this delineation is marked illicit substances as compared to more ‘societally acceptable’ or legal substances. It is possible that the women engaging in alcohol and cannabis use did not meet the threshold for a SUD, or stigma related to engagement in more socially acceptable substances may prevent individuals from accessing care or recognizing the need for treatment.

Based on our findings, there are specific actions that prisons and probation systems can take to better serve the population of Black women who use substances. First, implementing gender-responsive and culturally tailored programming is crucial. These systems should refer to and promote SUD treatment programs specifically designed for women, considering their unique needs and the intersectionality of their identities. Culturally sensitive approaches can enhance engagement and retention in treatment, leading to improved outcomes (Ehrmin, 2005; Greenfield et al., 2007; Guerrero & Andrews, 2011; Steinka-Fry et al., 2017). Second, enhancing access to comprehensive healthcare services is vital. Prisons and probation systems should ensure that Black women have access to healthcare services that address both their SUDs and other healthcare needs. This can involve collaborating with healthcare providers to deliver integrated care within correctional facilities or facilitating connections to community-based healthcare providers after release. By addressing the holistic health of Black women in these systems, their overall well-being can be improved, increasing the likelihood of successful reintegration into society and reduced recidivism rates.

There are several limitations to our analysis. As our results are limited to a small sample recruited from a single state, the results may not be generalizable to Black women living in other states. Also, we used drug use as a proxy for persons that need drug treatment; thus, not all women in our sample may need treatment services. However, subgroup analyses (not shown) among women who used drugs every day and among individuals who were not incarcerated during the study period were consistent the overall findings. Additionally, self-report and recall bias may be present; however, prior research has found criminal justice involved and other vulnerable populations recall of their behavior to be accurate (Darke, 1998; Napper et al., 2010). To further mitigate these biases, we utilized standardized assessment tools, including the Miami Health Services Utilization measures (Chitwood et al., 1999), which has been widely used in research settings. These tools are designed to elicit accurate information about service utilization. Another limitation of our study is the higher proportion of individuals lost to follow-up among those recruited from the prison setting compared to probation offices and the community sample. This discrepancy may introduce selection bias and should be considered when interpreting the generalizability of our findings. Importantly, the age of the data raises important considerations regarding the validity and generalizability of the results, as social and societal factors may have changed over time, such as the opioid epidemic. The impact of these changes on the applicability of the findings cannot be fully determined without further investigation or more recent data. While our study did not find a significant relationship between the number of days participants were free from incarceration following their release and treatment access^1^, future research may delve further into this aspect. Exploring the dynamics of service utilization during transitional periods and investigating the influence of support networks, community resources, and reentry programs could enhance our understanding of the barriers and facilitators to treatment engagement for justice-involved populations. Future research could delve into the specific transitional care services and discharge planning practices in Kentucky and their impact on treatment engagement outcomes for individuals returning from prison.

Nevertheless, the study focused on Black women across the criminal justice spectrum provides valuable insights into a specific time period, serving as a foundation for understanding historical trends and facilitating comparisons with more recent research. Future studies incorporating up-to-date data can enhance our understanding of the topic in the context of the evolving landscape of Black women in the criminal justice system and their SUD treatment engagement. It is important to acknowledge that while it is challenging to establish causality definitively in observational studies, our methodology provides a strong foundation for examining and understanding the temporal dynamics between services and SUD treatment among Black women who use substances. Additionally, it is important to consider that individuals involved in the criminal justice system face unique challenges and circumstances that may contribute to the higher attrition rate. Multiple factors such as transfers to other facilities, changes in parole or probation status, or difficulties in maintaining contact after release could all contribute to the increased loss to follow-up in this group. While we acknowledge this limitation, we also recognize the importance of understanding the potential impact it may have on the interpretation of our results. We encourage future research to explore strategies for minimizing loss to follow-up, particularly among justice-involved populations.

## Conclusion

In conclusion, this study provides valuable insights into the utilization of SUD treatment among Black women in different criminal justice-involved settings. The findings indicate a decrease in treatment utilization over time among Black women, highlighting the need to address retention challenges and disparities in accessing treatment. The role of the criminal justice system, including post-release monitoring and parole systems, is an important factor to consider when engaging Black women in SUD treatment. It is crucial to examine the settings frequented by this historically underserved group and expand efforts to reduce stigma and bias in these settings. The study also emphasizes the importance of integrating ED care with SUD treatment, as ED visits may serve as a signal of higher addiction severity or disadvantage among Black women. Enhancing collaboration between justice and treatment sectors, improving outreach efforts, and promoting screening and referral initiatives are key recommendations to improve access to SUD treatment for Black women who have SUD. Addressing structural racism, institutional distrust, and discriminatory policies, along with the integration of services and innovative outreach approaches, are essential in meeting the social and treatment needs of women while ensuring equitable access to necessary care.

## Data Availability

The dataset used and/or analyzed during the current study are available from the corresponding author on reasonable request.

## Footnotes

1 In our sensitivity analysis, we considered the inclusion of a time-varying measure for “time on the streets,“ which assessed the number of days participants were free from incarceration following their release for all 3 groups. However, we found that the effect size of the “time on the streets” variable was very small (0.001), and its inclusion did not substantially improve the models. Consequently, we made the decision to exclude these variables from the final analyses. Although the measure of “time on the streets” could have provided insights into treatment access during transitional periods, its exclusion was based on the lack of significant association and higher AIC/BIC values.

## Abbreviations

AFDC: Aid to Families with Dependent Children
AIC: Akaike’s information criterion
BIC: Bayesian information criterion
B-WISE: Black Women in the Study of Epidemics
ED: Emergency Department
GED: General Educational Development
RMSEA: Root mean squared error of approximation
TANF: Temporary Assistance for Needy Families
